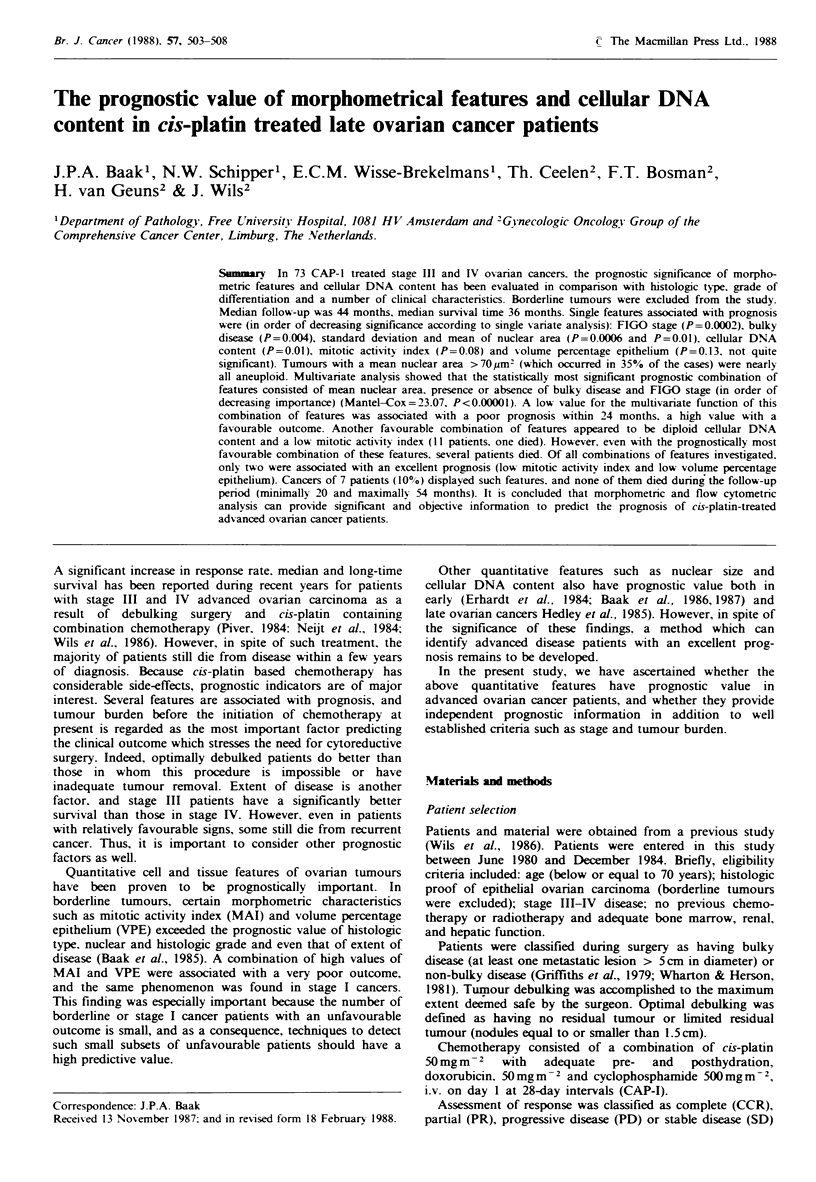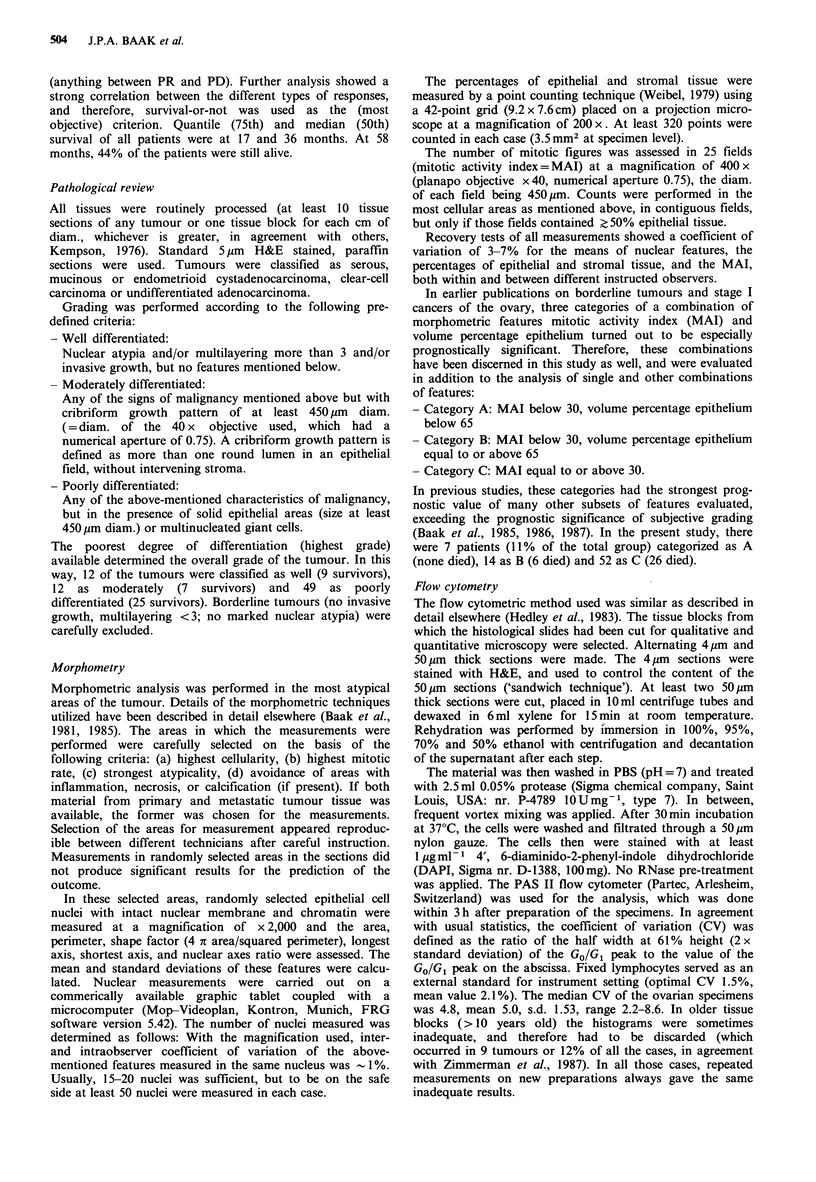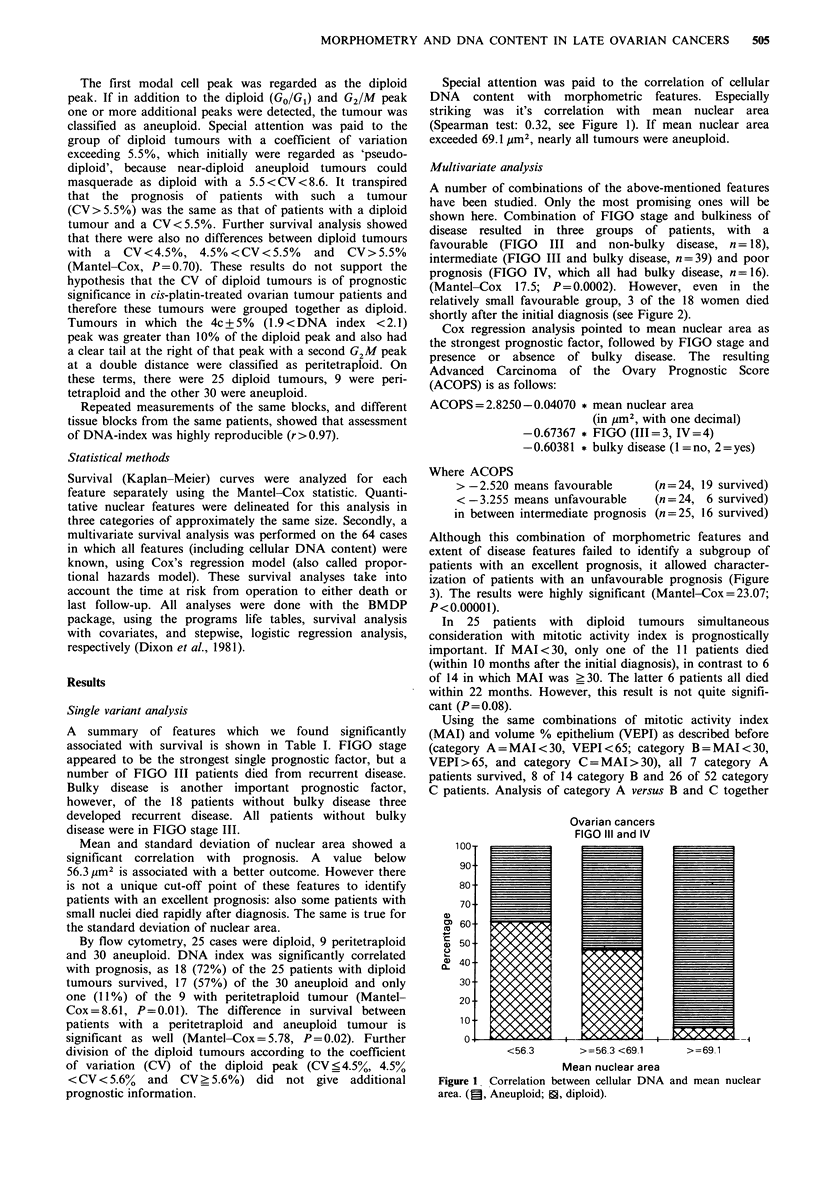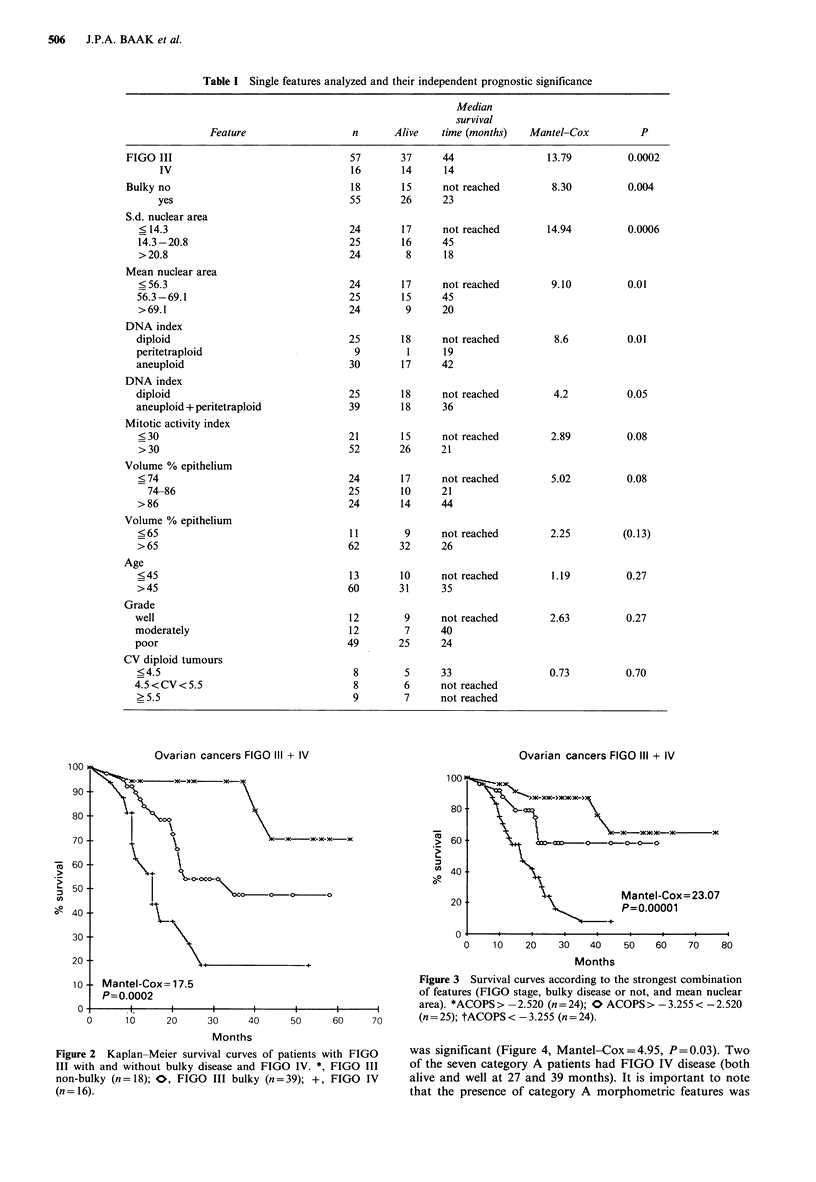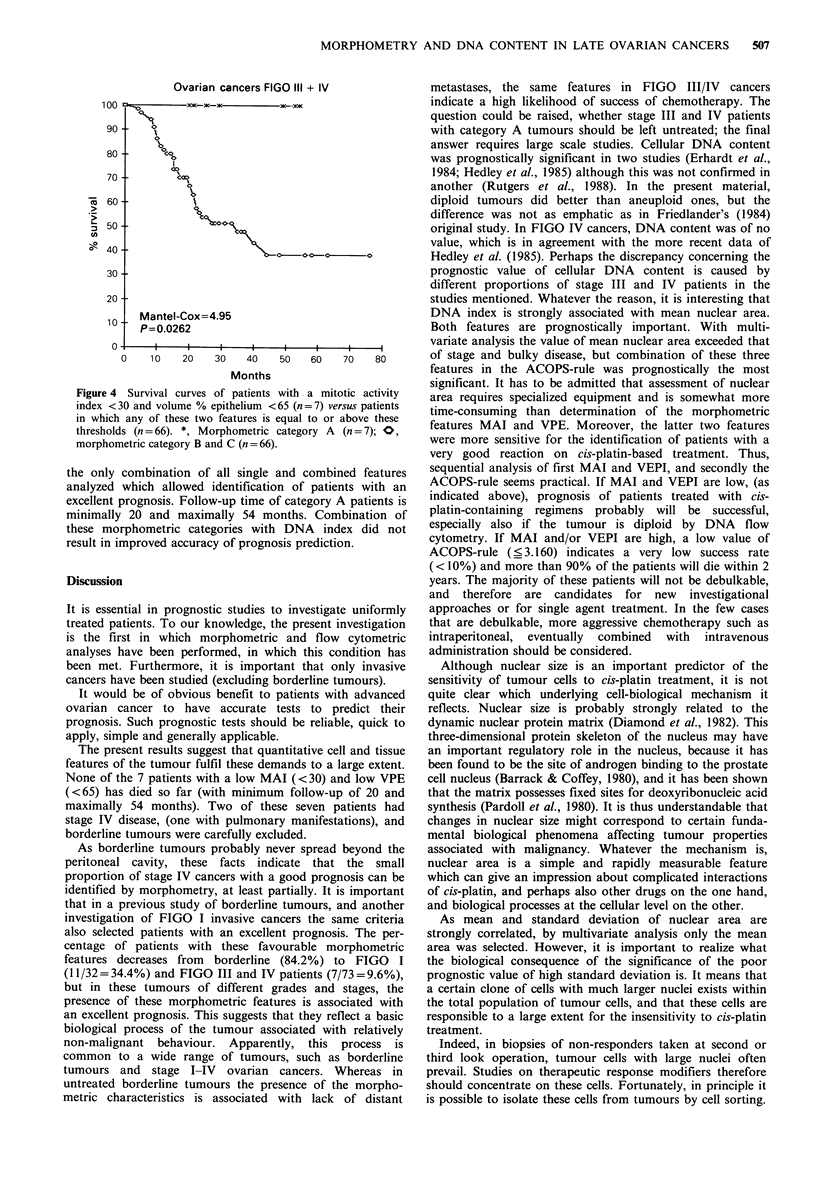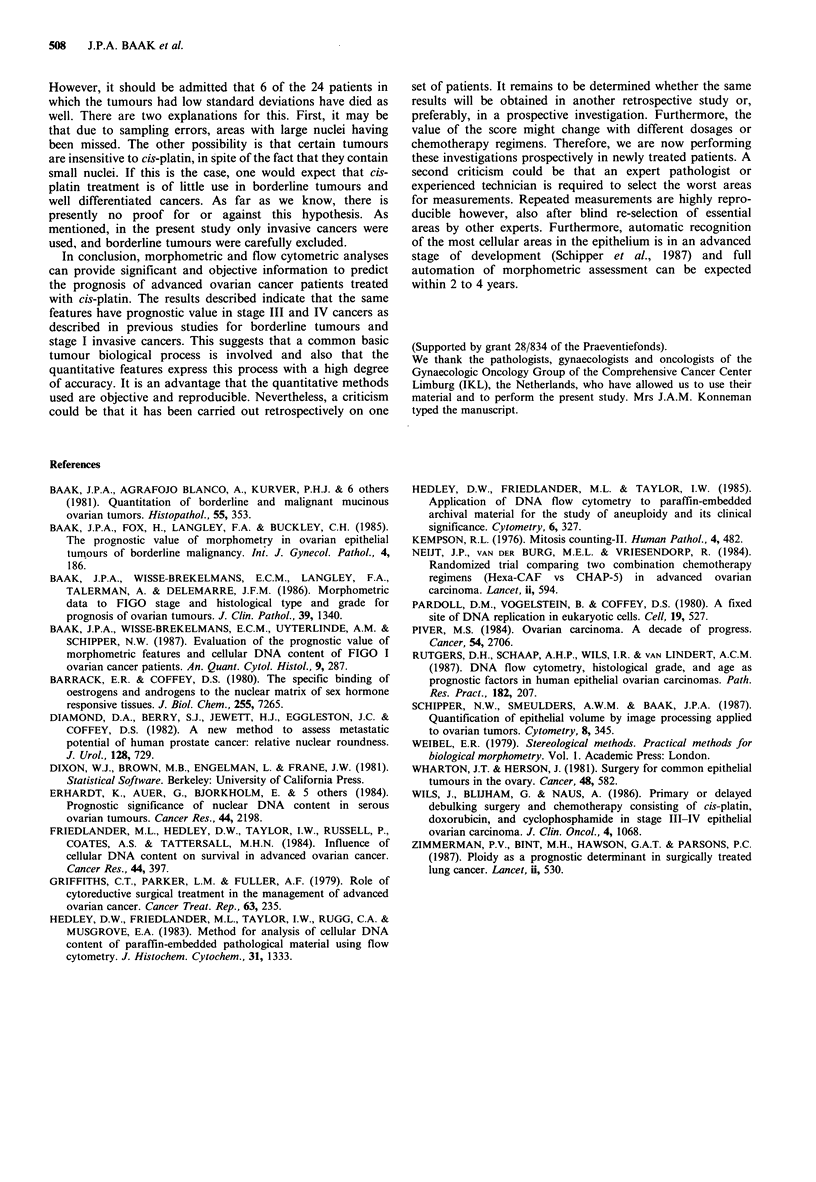# The prognostic value of morphometrical features and cellular DNA content in cis-platin treated late ovarian cancer patients.

**DOI:** 10.1038/bjc.1988.114

**Published:** 1988-05

**Authors:** J. P. Baak, N. W. Schipper, E. C. Wisse-Brekelmans, T. Ceelen, F. T. Bosman, H. van Geuns, J. Wils

**Affiliations:** Department of Pathology, Free University Hospital, Amsterdam, The Netherlands.

## Abstract

In 73 CAP-1 treated stage III and IV ovarian cancers, the prognostic significance of morphometric features and cellular DNA content has been evaluated in comparison with histologic type, grade of differentiation and a number of clinical characteristics. Borderline tumours were excluded from the study. Median follow-up was 44 months, median survival time 36 months. Single features associated with prognosis were (in order of decreasing significance according to single variate analysis): FIGO stage (P = 0.0002), bulky disease (P = 0.004), standard deviation and mean of nuclear area (P = 0.0006 and P = 0.01), cellular DNA content (P = 0.01), mitotic activity index (P = 0.08) and volume percentage epithelium (P = 0.13, not quite significant). Tumours with a mean nuclear area greater than 70 micron2 (which occurred in 35% of the cases) were nearly all aneuploid. Multivariate analysis showed that the statistically most significant prognostic combination of features consisted of mean nuclear area, presence or absence of bulky disease and FIGO stage (in order of decreasing importance) (Mantel-Cox = 23.07, P less than 0.00001). A low value for the multivariate function of this combination of features was associated with a poor prognosis within 24 months, a high value with a favourable outcome. Another favourable combination of features appeared to be diploid cellular DNA content and a low mitotic activity index (11 patients, one died). However, even with the prognostically most favourable combination of these features, several patients died. Of all combinations of features investigated, only two were associated with an excellent prognosis (low mitotic activity index and low volume percentage epithelium). Cancers of 7 patients (10%) displayed such features, and none of them died during the follow-up period (minimally 20 and maximally 54 months). It is concluded that morphometric and flow cytometric analysis can provide significant and objective information to predict the prognosis of cis-platin-treated advanced ovarian cancer patients.


Br. J. Cancer (1988), 57, 503-508 © The Macmillan Press Ltd., 1988

### The prognostic value of morphometrical features and cellular DNA content in *cis*-platin treated late ovarian cancer patients

J.P.A. Baak[1], N.W. Schipper[1], E.C.M. Wisse-Brekelmans[1], Th. Ceelen[2], F.T. Bosman[2], H. van Geuns[2] & J. Wils[2]

[1]*Department of Pathology, Free University Hospital, 1081 HV Amsterdam and* [2]*Gynecologic Oncology Group of the Comprehensive Cancer Center, Limburg, The Netherlands.*

**Summary** In 73 CAP-1 treated stage III and IV ovarian cancers, the prognostic significance of morphometric features and cellular DNA content has been evaluated in comparison with histologic type, grade of differentiation and a number of clinical characteristics. Borderline tumours were excluded from the study. Median follow-up was 44 months, median survival time 36 months. Single features associated with prognosis were (in order of decreasing significance according to single variate analysis): FIGO stage ($P=0.0002$), bulky disease ($P=0.004$), standard deviation and mean of nuclear area ($P=0.0006$ and $P=0.01$), cellular DNA content ($P=0.01$), mitotic activity index ($P=0.08$) and volume percentage epithelium ($P=0.13$, not quite significant). Tumours with a mean nuclear area $>70\,\mu m^2$ (which occurred in 35% of the cases) were nearly all aneuploid. Multivariate analysis showed that the statistically most significant prognostic combination of features consisted of mean nuclear area, presence or absence of bulky disease and FIGO stage (in order of decreasing importance) (Mantel–Cox = 23.07, $P<0.00001$). A low value for the multivariate function of this combination of features was associated with a poor prognosis within 24 months, a high value with a favourable outcome. Another favourable combination of features appeared to be diploid cellular DNA content and a low mitotic activity index (11 patients, one died). However, even with the prognostically most favourable combination of these features, several patients died. Of all combinations of features investigated, only two were associated with an excellent prognosis (low mitotic activity index and low volume percentage epithelium). Cancers of 7 patients (10%) displayed such features, and none of them died during the follow-up period (minimally 20 and maximally 54 months). It is concluded that morphometric and flow cytometric analysis can provide significant and objective information to predict the prognosis of *cis*-platin-treated advanced ovarian cancer patients.

A significant increase in response rate, median and long-time survival has been reported during recent years for patients with stage III and IV advanced ovarian carcinoma as a result of debulking surgery and *cis*-platin containing combination chemotherapy (Piver, 1984: Neijt *et al.*, 1984; Wils *et al.*, 1986). However, in spite of such treatment, the majority of patients still die from disease within a few years of diagnosis. Because *cis*-platin based chemotherapy has considerable side-effects, prognostic indicators are of major interest. Several features are associated with prognosis, and tumour burden before the initiation of chemotherapy at present is regarded as the most important factor predicting the clinical outcome which stresses the need for cytoreductive surgery. Indeed, optimally debulked patients do better than those in whom this procedure is impossible or have inadequate tumour removal. Extent of disease is another factor, and stage III patients have a significantly better survival than those in stage IV. However, even in patients with relatively favourable signs, some still die from recurrent cancer. Thus, it is important to consider other prognostic factors as well.

Quantitative cell and tissue features of ovarian tumours have been proven to be prognostically important. In borderline tumours, certain morphometric characteristics such as mitotic activity index (MAI) and volume percentage epithelium (VPE) exceeded the prognostic value of histologic type, nuclear and histologic grade and even that of extent of disease (Baak *et al.*, 1985). A combination of high values of MAI and VPE were associated with a very poor outcome, and the same phenomenon was found in stage I cancers. This finding was especially important because the number of borderline or stage I cancer patients with an unfavourable outcome is small, and as a consequence, techniques to detect such small subsets of unfavourable patients should have a high predictive value.

Correspondence: J.P.A. Baak
Received 13 November 1987; and in revised form 18 February 1988.

Other quantitative features such as nuclear size and cellular DNA content also have prognostic value both in early (Erhardt *et al.*, 1984; Baak *et al.*, 1986, 1987) and late ovarian cancers Hedley *et al.*, 1985). However, in spite of the significance of these findings, a method which can identify advanced disease patients with an excellent prognosis remains to be developed.

In the present study, we have ascertained whether the above quantitative features have prognostic value in advanced ovarian cancer patients, and whether they provide independent prognostic information in addition to well established criteria such as stage and tumour burden.

### Materials and methods

### Patient selection

Patients and material were obtained from a previous study (Wils *et al.*, 1986). Patients were entered in this study between June 1980 and December 1984. Briefly, eligibility criteria included: age (below or equal to 70 years); histologic proof of epithelial ovarian carcinoma (borderline tumours were excluded); stage III–IV disease; no previous chemotherapy or radiotherapy and adequate bone marrow, renal, and hepatic function.

Patients were classified during surgery as having bulky disease (at least one metastatic lesion > 5 cm in diameter) or non-bulky disease (Griffiths *et al.*, 1979; Wharton & Herson, 1981). Tumour debulking was accomplished to the maximum extent deemed safe by the surgeon. Optimal debulking was defined as having no residual tumour or limited residual tumour (nodules equal to or smaller than 1.5 cm).

Chemotherapy consisted of a combination of *cis*-platin $50\,mg\,m^{-2}$ with adequate pre- and posthydration, doxorubicin, $50\,mg\,m^{-2}$ and cyclophosphamide $500\,mg\,m^{-2}$, i.v. on day 1 at 28-day intervals (CAP-I).

Assessment of response was classified as complete (CCR), partial (PR), progressive disease (PD) or stable disease (SD)

(anything between PR and PD). Further analysis showed a strong correlation between the different types of responses, and therefore, survival-or-not was used as the (most objective) criterion. Quantile (75th) and median (50th) survival of all patients were at 17 and 36 months. At 58 months, 44% of the patients were still alive.

*Pathological review*

All tissues were routinely processed (at least 10 tissue sections of any tumour or one tissue block for each cm of diam., whichever is greater, in agreement with others, Kempson, 1976). Standard 5 μm H&E stained, paraffin sections were used. Tumours were classified as serous, mucinous or endometrioid cystadenocarcinoma, clear-cell carcinoma or undifferentiated adenocarcinoma.

Grading was performed according to the following predefined criteria:

- Well differentiated:
  Nuclear atypia and/or multilayering more than 3 and/or invasive growth, but no features mentioned below.
- Moderately differentiated:
  Any of the signs of malignancy mentioned above but with cribriform growth pattern of at least 450 μm diam. (= diam. of the 40× objective used, which had a numerical aperture of 0.75). A cribriform growth pattern is defined as more than one round lumen in an epithelial field, without intervening stroma.
- Poorly differentiated:
  Any of the above-mentioned characteristics of malignancy, but in the presence of solid epithelial areas (size at least 450 μm diam.) or multinucleated giant cells.

The poorest degree of differentiation (highest grade) available determined the overall grade of the tumour. In this way, 12 of the tumours were classified as well (9 survivors), 12 as moderately (7 survivors) and 49 as poorly differentiated (25 survivors). Borderline tumours (no invasive growth, multilayering $<3$; no marked nuclear atypia) were carefully excluded.

*Morphometry*

Morphometric analysis was performed in the most atypical areas of the tumour. Details of the morphometric techniques utilized have been described in detail elsewhere (Baak *et al.*, 1981, 1985). The areas in which the measurements were performed were carefully selected on the basis of the following criteria: (a) highest cellularity, (b) highest mitotic rate, (c) strongest atypicality, (d) avoidance of areas with inflammation, necrosis, or calcification (if present). If both material from primary and metastatic tumour tissue was available, the former was chosen for the measurements. Selection of the areas for measurement appeared reproducible between different technicians after careful instruction. Measurements in randomly selected areas in the sections did not produce significant results for the prediction of the outcome.

In these selected areas, randomly selected epithelial cell nuclei with intact nuclear membrane and chromatin were measured at a magnification of ×2,000 and the area, perimeter, shape factor (4 π area/squared perimeter), longest axis, shortest axis, and nuclear axes ratio were assessed. The mean and standard deviations of these features were calculated. Nuclear measurements were carried out on a commerically available graphic tablet coupled with a microcomputer (Mop–Videoplan, Kontron, Munich, FRG software version 5.42). The number of nuclei measured was determined as follows: With the magnification used, inter- and intraobserver coefficient of variation of the above-mentioned features measured in the same nucleus was ~1%. Usually, 15–20 nuclei was sufficient, but to be on the safe side at least 50 nuclei were measured in each case.

The percentages of epithelial and stromal tissue were measured by a point counting technique (Weibel, 1979) using a 42-point grid (9.2×7.6 cm) placed on a projection microscope at a magnification of 200×. At least 320 points were counted in each case (3.5 $mm^2$ at specimen level).

The number of mitotic figures was assessed in 25 fields (mitotic activity index = MAI) at a magnification of 400× (planapo objective ×40, numerical aperture 0.75), the diam. of each field being 450 μm. Counts were performed in the most cellular areas as mentioned above, in contiguous fields, but only if those fields contained $\gtrsim 50\%$ epithelial tissue.

Recovery tests of all measurements showed a coefficient of variation of 3–7% for the means of nuclear features, the percentages of epithelial and stromal tissue, and the MAI, both within and between different instructed observers.

In earlier publications on borderline tumours and stage I cancers of the ovary, three categories of a combination of morphometric features mitotic activity index (MAI) and volume percentage epithelium turned out to be especially prognostically significant. Therefore, these combinations have been discerned in this study as well, and were evaluated in addition to the analysis of single and other combinations of features:

- Category A: MAI below 30, volume percentage epithelium below 65
- Category B: MAI below 30, volume percentage epithelium equal to or above 65
- Category C: MAI equal to or above 30.

In previous studies, these categories had the strongest prognostic value of many other subsets of features evaluated, exceeding the prognostic significance of subjective grading (Baak *et al.*, 1985, 1986, 1987). In the present study, there were 7 patients (11% of the total group) categorized as A (none died), 14 as B (6 died) and 52 as C (26 died).

*Flow cytometry*

The flow cytometric method used was similar as described in detail elsewhere (Hedley *et al.*, 1983). The tissue blocks from which the histological slides had been cut for qualitative and quantitative microscopy were selected. Alternating 4 μm and 50 μm thick sections were made. The 4 μm sections were stained with H&E, and used to control the content of the 50 μm sections ('sandwich technique'). At least two 50 μm thick sections were cut, placed in 10 ml centrifuge tubes and dewaxed in 6 ml xylene for 15 min at room temperature. Rehydration was performed by immersion in 100%, 95%, 70% and 50% ethanol with centrifugation and decantation of the supernatant after each step.

The material was then washed in PBS (pH = 7) and treated with 2.5 ml 0.05% protease (Sigma chemical company, Saint Louis, USA: nr. P-4789 10 U $mg^{-1}$, type 7). In between, frequent vortex mixing was applied. After 30 min incubation at 37°C, the cells were washed and filtrated through a 50 μm nylon gauze. The cells then were stained with at least 1 μg $ml^{-1}$ 4′, 6-diaminido-2-phenyl-indole dihydrochloride (DAPI, Sigma nr. D-1388, 100 mg). No RNase pre-treatment was applied. The PAS II flow cytometer (Partec, Arlesheim, Switzerland) was used for the analysis, which was done within 3 h after preparation of the specimens. In agreement with usual statistics, the coefficient of variation (CV) was defined as the ratio of the half width at 61% height (2× standard deviation) of the $G_0/G_1$ peak to the value of the $G_0/G_1$ peak on the abscissa. Fixed lymphocytes served as an external standard for instrument setting (optimal CV 1.5%, mean value 2.1%). The median CV of the ovarian specimens was 4.8, mean 5.0, s.d. 1.53, range 2.2–8.6. In older tissue blocks (>10 years old) the histograms were sometimes inadequate, and therefore had to be discarded (which occurred in 9 tumours or 12% of all the cases, in agreement with Zimmerman *et al.*, 1987). In all those cases, repeated measurements on new preparations always gave the same inadequate results.

The first modal cell peak was regarded as the diploid peak. If in addition to the diploid ($G_0/G_1$) and $G_2/M$ peak one or more additional peaks were detected, the tumour was classified as aneuploid. Special attention was paid to the group of diploid tumours with a coefficient of variation exceeding 5.5%, which initially were regarded as 'pseudo-diploid', because near-diploid aneuploid tumours could masquerade as diploid with a $5.5<CV<8.6$. It transpired that the prognosis of patients with such a tumour ($CV>5.5\%$) was the same as that of patients with a diploid tumour and a $CV<5.5\%$. Further survival analysis showed that there were also no differences between diploid tumours with a $CV<4.5\%$, $4.5\%<CV<5.5\%$ and $CV>5.5\%$ (Mantel–Cox, $P=0.70$). These results do not support the hypothesis that the CV of diploid tumours is of prognostic significance in *cis*-platin-treated ovarian tumour patients and therefore these tumours were grouped together as diploid. Tumours in which the $4c\pm5\%$ ($1.9<$DNA index $<2.1$) peak was greater than 10% of the diploid peak and also had a clear tail at the right of that peak with a second $G_2M$ peak at a double distance were classified as peritetraploid. On these terms, there were 25 diploid tumours, 9 were peritetraploid and the other 30 were aneuploid.

Repeated measurements of the same blocks, and different tissue blocks from the same patients, showed that assessment of DNA-index was highly reproducible ($r>0.97$).

*Statistical methods*

Survival (Kaplan–Meier) curves were analyzed for each feature separately using the Mantel–Cox statistic. Quantitative nuclear features were delineated for this analysis in three categories of approximately the same size. Secondly, a multivariate survival analysis was performed on the 64 cases in which all features (including cellular DNA content) were known, using Cox's regression model (also called proportional hazards model). These survival analyses take into account the time at risk from operation to either death or last follow-up. All analyses were done with the BMDP package, using the programs life tables, survival analysis with covariates, and stepwise, logistic regression analysis, respectively (Dixon *et al.*, 1981).

### Results

*Single variant analysis*

A summary of features which we found significantly associated with survival is shown in Table I. FIGO stage appeared to be the strongest single prognostic factor, but a number of FIGO III patients died from recurrent disease. Bulky disease is another important prognostic factor, however, of the 18 patients without bulky disease three developed recurrent disease. All patients without bulky disease were in FIGO stage III.

Mean and standard deviation of nuclear area showed a significant correlation with prognosis. A value below 56.3 $\mu m^2$ is associated with a better outcome. However there is not a unique cut-off point of these features to identify patients with an excellent prognosis: also some patients with small nuclei died rapidly after diagnosis. The same is true for the standard deviation of nuclear area.

By flow cytometry, 25 cases were diploid, 9 peritetraploid and 30 aneuploid. DNA index was significantly correlated with prognosis, as 18 (72%) of the 25 patients with diploid tumours survived, 17 (57%) of the 30 aneuploid and only one (11%) of the 9 with peritetraploid tumour (Mantel–Cox = 8.61, $P=0.01$). The difference in survival between patients with a peritetraploid and aneuploid tumour is significant as well (Mantel–Cox = 5.78, $P=0.02$). Further division of the diploid tumours according to the coefficient of variation (CV) of the diploid peak ($CV\leqq4.5\%$, $4.5\%<CV<5.6\%$ and $CV\geqq5.6\%$) did not give additional prognostic information.

Special attention was paid to the correlation of cellular DNA content with morphometric features. Especially striking was it's correlation with mean nuclear area (Spearman test: 0.32, see Figure 1). If mean nuclear area exceeded 69.1 $\mu m^2$, nearly all tumours were aneuploid.

*Multivariate analysis*

A number of combinations of the above-mentioned features have been studied. Only the most promising ones will be shown here. Combination of FIGO stage and bulkiness of disease resulted in three groups of patients, with a favourable (FIGO III and non-bulky disease, $n=18$), intermediate (FIGO III and bulky disease, $n=39$) and poor prognosis (FIGO IV, which all had bulky disease, $n=16$). (Mantel–Cox 17.5; $P=0.0002$). However, even in the relatively small favourable group, 3 of the 18 women died shortly after the initial diagnosis (see Figure 2).

Cox regression analysis pointed to mean nuclear area as the strongest prognostic factor, followed by FIGO stage and presence or absence of bulky disease. The resulting Advanced Carcinoma of the Ovary Prognostic Score (ACOPS) is as follows:

ACOPS = 2.8250 − 0.04070 * mean nuclear area (in $\mu m^2$, with one decimal)

−0.67367 * FIGO (III = 3, IV = 4)

−0.60381 * bulky disease (1 = no, 2 = yes)

Where ACOPS

> −2.520 means favourable ($n=24$, 19 survived)

< −3.255 means unfavourable ($n=24$, 6 survived)

in between intermediate prognosis ($n=25$, 16 survived)

Although this combination of morphometric features and extent of disease features failed to identify a subgroup of patients with an excellent prognosis, it allowed characterization of patients with an unfavourable prognosis (Figure 3). The results were highly significant (Mantel–Cox = 23.07; $P<0.00001$).

In 25 patients with diploid tumours simultaneous consideration with mitotic activity index is prognostically important. If MAI < 30, only one of the 11 patients died (within 10 months after the initial diagnosis), in contrast to 6 of 14 in which MAI was ≧30. The latter 6 patients all died within 22 months. However, this result is not quite significant ($P=0.08$).

Using the same combinations of mitotic activity index (MAI) and volume % epithelium (VEPI) as described before (category A = MAI < 30, VEPI < 65; category B = MAI < 30, VEPI > 65, and category C = MAI > 30), all 7 category A patients survived, 8 of 14 category B and 26 of 52 category C patients. Analysis of category A *versus* B and C together

**Figure 1** Correlation between cellular DNA and mean nuclear area. (▤, Aneuploid; ▨, diploid).

**Table I** Single features analyzed and their independent prognostic significance

| *Feature* | *n* | *Alive* | *Median survival time (months)* | *Mantel–Cox* | *P* |
|---|---|---|---|---|---|
| FIGO III | 57 | 37 | 44 | 13.79 | 0.0002 |
| IV | 16 | 14 | 14 | | |
| Bulky no | 18 | 15 | not reached | 8.30 | 0.004 |
| yes | 55 | 26 | 23 | | |
| S.d. nuclear area | | | | | |
| ≦14.3 | 24 | 17 | not reached | 14.94 | 0.0006 |
| 14.3–20.8 | 25 | 16 | 45 | | |
| >20.8 | 24 | 8 | 18 | | |
| Mean nuclear area | | | | | |
| ≦56.3 | 24 | 17 | not reached | 9.10 | 0.01 |
| 56.3–69.1 | 25 | 15 | 45 | | |
| >69.1 | 24 | 9 | 20 | | |
| DNA index | | | | | |
| diploid | 25 | 18 | not reached | 8.6 | 0.01 |
| peritetraploid | 9 | 1 | 19 | | |
| aneuploid | 30 | 17 | 42 | | |
| DNA index | | | | | |
| diploid | 25 | 18 | not reached | 4.2 | 0.05 |
| aneuploid + peritetraploid | 39 | 18 | 36 | | |
| Mitotic activity index | | | | | |
| ≦30 | 21 | 15 | not reached | 2.89 | 0.08 |
| >30 | 52 | 26 | 21 | | |
| Volume % epithelium | | | | | |
| ≦74 | 24 | 17 | not reached | 5.02 | 0.08 |
| 74–86 | 25 | 10 | 21 | | |
| >86 | 24 | 14 | 44 | | |
| Volume % epithelium | | | | | |
| ≦65 | 11 | 9 | not reached | 2.25 | (0.13) |
| >65 | 62 | 32 | 26 | | |
| Age | | | | | |
| ≦45 | 13 | 10 | not reached | 1.19 | 0.27 |
| >45 | 60 | 31 | 35 | | |
| Grade | | | | | |
| well | 12 | 9 | not reached | 2.63 | 0.27 |
| moderately | 12 | 7 | 40 | | |
| poor | 49 | 25 | 24 | | |
| CV diploid tumours | | | | | |
| ≦4.5 | 8 | 5 | 33 | 0.73 | 0.70 |
| 4.5<CV<5.5 | 8 | 6 | not reached | | |
| ≧5.5 | 9 | 7 | not reached | | |

**Figure 2** Kaplan–Meier survival curves of patients with FIGO III with and without bulky disease and FIGO IV. *, FIGO III non-bulky ($n=18$); O, FIGO III bulky ($n=39$); +, FIGO IV ($n=16$).

**Figure 3** Survival curves according to the strongest combination of features (FIGO stage, bulky disease or not, and mean nuclear area). *ACOPS > −2.520 ($n=24$); O ACOPS > −3.255 < −2.520 ($n=25$); †ACOPS < −3.255 ($n=24$).

was significant (Figure 4, Mantel–Cox = 4.95, $P=0.03$). Two of the seven category A patients had FIGO IV disease (both alive and well at 27 and 39 months). It is important to note that the presence of category A morphometric features was

**Figure 4** Survival curves of patients with a mitotic activity index <30 and volume % epithelium <65 ($n=7$) *versus* patients in which any of these two features is equal to or above these thresholds ($n=66$). *, Morphometric category A ($n=7$); O, morphometric category B and C ($n=66$).

the only combination of all single and combined features analyzed which allowed identification of patients with an excellent prognosis. Follow-up time of category A patients is minimally 20 and maximally 54 months. Combination of these morphometric categories with DNA index did not result in improved accuracy of prognosis prediction.

### Discussion

It is essential in prognostic studies to investigate uniformly treated patients. To our knowledge, the present investigation is the first in which morphometric and flow cytometric analyses have been performed, in which this condition has been met. Furthermore, it is important that only invasive cancers have been studied (excluding borderline tumours).

It would be of obvious benefit to patients with advanced ovarian cancer to have accurate tests to predict their prognosis. Such prognostic tests should be reliable, quick to apply, simple and generally applicable.

The present results suggest that quantitative cell and tissue features of the tumour fulfil these demands to a large extent. None of the 7 patients with a low MAI (<30) and low VPE (<65) has died so far (with minimum follow-up of 20 and maximally 54 months). Two of these seven patients had stage IV disease, (one with pulmonary manifestations), and borderline tumours were carefully excluded.

As borderline tumours probably never spread beyond the peritoneal cavity, these facts indicate that the small proportion of stage IV cancers with a good prognosis can be identified by morphometry, at least partially. It is important that in a previous study of borderline tumours, and another investigation of FIGO I invasive cancers the same criteria also selected patients with an excellent prognosis. The percentage of patients with these favourable morphometric features decreases from borderline (84.2%) to FIGO I (11/32=34.4%) and FIGO III and IV patients (7/73=9.6%), but in these tumours of different grades and stages, the presence of these morphometric features is associated with an excellent prognosis. This suggests that they reflect a basic biological process of the tumour associated with relatively non-malignant behaviour. Apparently, this process is common to a wide range of tumours, such as borderline tumours and stage I–IV ovarian cancers. Whereas in untreated borderline tumours the presence of the morphometric characteristics is associated with lack of distant metastases, the same features in FIGO III/IV cancers indicate a high likelihood of success of chemotherapy. The question could be raised, whether stage III and IV patients with category A tumours should be left untreated; the final answer requires large scale studies. Cellular DNA content was prognostically significant in two studies (Erhardt *et al.*, 1984; Hedley *et al.*, 1985) although this was not confirmed in another (Rutgers *et al.*, 1988). In the present material, diploid tumours did better than aneuploid ones, but the difference was not as emphatic as in Friedlander's (1984) original study. In FIGO IV cancers, DNA content was of no value, which is in agreement with the more recent data of Hedley *et al.* (1985). Perhaps the discrepancy concerning the prognostic value of cellular DNA content is caused by different proportions of stage III and IV patients in the studies mentioned. Whatever the reason, it is interesting that DNA index is strongly associated with mean nuclear area. Both features are prognostically important. With multivariate analysis the value of mean nuclear area exceeded that of stage and bulky disease, but combination of these three features in the ACOPS-rule was prognostically the most significant. It has to be admitted that assessment of nuclear area requires specialized equipment and is somewhat more time-consuming than determination of the morphometric features MAI and VPE. Moreover, the latter two features were more sensitive for the identification of patients with a very good reaction on *cis*-platin-based treatment. Thus, sequential analysis of first MAI and VEPI, and secondly the ACOPS-rule seems practical. If MAI and VEPI are low, (as indicated above), prognosis of patients treated with *cis*-platin-containing regimens probably will be successful, especially also if the tumour is diploid by DNA flow cytometry. If MAI and/or VEPI are high, a low value of ACOPS-rule (≦3.160) indicates a very low success rate (<10%) and more than 90% of the patients will die within 2 years. The majority of these patients will not be debulkable, and therefore are candidates for new investigational approaches or for single agent treatment. In the few cases that are debulkable, more aggressive chemotherapy such as intraperitoneal, eventually combined with intravenous administration should be considered.

Although nuclear size is an important predictor of the sensitivity of tumour cells to *cis*-platin treatment, it is not quite clear which underlying cell-biological mechanism it reflects. Nuclear size is probably strongly related to the dynamic nuclear protein matrix (Diamond *et al.*, 1982). This three-dimensional protein skeleton of the nucleus may have an important regulatory role in the nucleus, because it has been found to be the site of androgen binding to the prostate cell nucleus (Barrack & Coffey, 1980), and it has been shown that the matrix possesses fixed sites for deoxyribonucleic acid synthesis (Pardoll *et al.*, 1980). It is thus understandable that changes in nuclear size might correspond to certain fundamental biological phenomena affecting tumour properties associated with malignancy. Whatever the mechanism is, nuclear area is a simple and rapidly measurable feature which can give an impression about complicated interactions of *cis*-platin, and perhaps also other drugs on the one hand, and biological processes at the cellular level on the other.

As mean and standard deviation of nuclear area are strongly correlated, by multivariate analysis only the mean area was selected. However, it is important to realize what the biological consequence of the significance of the poor prognostic value of high standard deviation is. It means that a certain clone of cells with much larger nuclei exists within the total population of tumour cells, and that these cells are responsible to a large extent for the insensitivity to *cis*-platin treatment.

Indeed, in biopsies of non-responders taken at second or third look operation, tumour cells with large nuclei often prevail. Studies on therapeutic response modifiers therefore should concentrate on these cells. Fortunately, in principle it is possible to isolate these cells from tumours by cell sorting.

However, it should be admitted that 6 of the 24 patients in which the tumours had low standard deviations have died as well. There are two explanations for this. First, it may be that due to sampling errors, areas with large nuclei having been missed. The other possibility is that certain tumours are insensitive to *cis*-platin, in spite of the fact that they contain small nuclei. If this is the case, one would expect that *cis*-platin treatment is of little use in borderline tumours and well differentiated cancers. As far as we know, there is presently no proof for or against this hypothesis. As mentioned, in the present study only invasive cancers were used, and borderline tumours were carefully excluded.

In conclusion, morphometric and flow cytometric analyses can provide significant and objective information to predict the prognosis of advanced ovarian cancer patients treated with *cis*-platin. The results described indicate that the same features have prognostic value in stage III and IV cancers as described in previous studies for borderline tumours and stage I invasive cancers. This suggests that a common basic tumour biological process is involved and also that the quantitative features express this process with a high degree of accuracy. It is an advantage that the quantitative methods used are objective and reproducible. Nevertheless, a criticism could be that it has been carried out retrospectively on one set of patients. It remains to be determined whether the same results will be obtained in another retrospective study or, preferably, in a prospective investigation. Furthermore, the value of the score might change with different dosages or chemotherapy regimens. Therefore, we are now performing these investigations prospectively in newly treated patients. A second criticism could be that an expert pathologist or experienced technician is required to select the worst areas for measurements. Repeated measurements are highly reproducible however, also after blind re-selection of essential areas by other experts. Furthermore, automatic recognition of the most cellular areas in the epithelium is in an advanced stage of development (Schipper *et al.*, 1987) and full automation of morphometric assessment can be expected within 2 to 4 years.

(Supported by grant 28/834 of the Praeventiefonds).
We thank the pathologists, gynaecologists and oncologists of the Gynaecologic Oncology Group of the Comprehensive Cancer Center Limburg (IKL), the Netherlands, who have allowed us to use their material and to perform the present study. Mrs J.A.M. Konneman typed the manuscript.

### References

BAAK, J.P.A., AGRAFOJO BLANCO, A., KURVER, P.H.J. & 6 others (1981). Quantitation of borderline and malignant mucinous ovarian tumors. *Histopathol.*, **55**, 353.

BAAK, J.P.A., FOX, H., LANGLEY, F.A. & BUCKLEY, C.H. (1985). The prognostic value of morphometry in ovarian epithelial tumours of borderline malignancy. *Int. J. Gynecol. Pathol.*, **4**, 186.

BAAK, J.P.A., WISSE-BREKELMANS, E.C.M., LANGLEY, F.A., TALERMAN, A. & DELEMARRE, J.F.M. (1986). Morphometric data to FIGO stage and histological type and grade for prognosis of ovarian tumours. *J. Clin. Pathol.*, **39**, 1340.

BAAK, J.P.A., WISSE-BREKELMANS, E.C.M., UYTERLINDE, A.M. & SCHIPPER, N.W. (1987). Evaluation of the prognostic value of morphometric features and cellular DNA content of FIGO I ovarian cancer patients. *An. Quant. Cytol. Histol.*, **9**, 287.

BARRACK, E.R. & COFFEY, D.S. (1980). The specific binding of oestrogens and androgens to the nuclear matrix of sex hormone responsive tissues. *J. Biol. Chem.*, **255**, 7265.

DIAMOND, D.A., BERRY, S.J., JEWETT, H.J., EGGLESTON, J.C. & COFFEY, D.S. (1982). A new method to assess metastatic potential of human prostate cancer: relative nuclear roundness. *J. Urol.*, **128**, 729.

DIXON, W.J., BROWN, M.B., ENGELMAN, L. & FRANE, J.W. (1981). *Statistical Software*. Berkeley: University of California Press.

ERHARDT, K., AUER, G., BJORKHOLM, E. & 5 others (1984). Prognostic significance of nuclear DNA content in serous ovarian tumours. *Cancer Res.*, **44**, 2198.

FRIEDLANDER, M.L., HEDLEY, D.W., TAYLOR, I.W., RUSSELL, P., COATES, A.S. & TATTERSALL, M.H.N. (1984). Influence of cellular DNA content on survival in advanced ovarian cancer. *Cancer Res.*, **44**, 397.

GRIFFITHS, C.T., PARKER, L.M. & FULLER, A.F. (1979). Role of cytoreductive surgical treatment in the management of advanced ovarian cancer. *Cancer Treat. Rep.*, **63**, 235.

HEDLEY, D.W., FRIEDLANDER, M.L., TAYLOR, I.W., RUGG, C.A. & MUSGROVE, E.A. (1983). Method for analysis of cellular DNA content of paraffin-embedded pathological material using flow cytometry. *J. Histochem. Cytochem.*, **31**, 1333.

HEDLEY, D.W., FRIEDLANDER, M.L. & TAYLOR, I.W. (1985). Application of DNA flow cytometry to paraffin-embedded archival material for the study of aneuploidy and its clinical significance. *Cytometry*, **6**, 327.

KEMPSON, R.L. (1976). Mitosis counting-II. *Human Pathol.*, **4**, 482.

NEIJT, J.P., VAN DER BURG, M.E.L. & VRIESENDORP, R. (1984). Randomized trial comparing two combination chemotherapy regimens (Hexa-CAF vs CHAP-5) in advanced ovarian carcinoma. *Lancet*, **ii**, 594.

PARDOLL, D.M., VOGELSTEIN, B. & COFFEY, D.S. (1980). A fixed site of DNA replication in eukaryotic cells. *Cell*, **19**, 527.

PIVER, M.S. (1984). Ovarian carcinoma. A decade of progress. *Cancer*, **54**, 2706.

RUTGERS, D.H., SCHAAP, A.H.P., WILS, I.R. & VAN LINDERT, A.C.M. (1987). DNA flow cytometry, histological grade, and age as prognostic factors in human epithelial ovarian carcinomas. *Path. Res. Pract.*, **182**, 207.

SCHIPPER, N.W., SMEULDERS, A.W.M. & BAAK, J.P.A. (1987). Quantification of epithelial volume by image processing applied to ovarian tumors. *Cytometry*, **8**, 345.

WEIBEL, E.R. (1979). *Stereological methods. Practical methods for biological morphometry*. Vol. 1. Academic Press: London.

WHARTON, J.T. & HERSON, J. (1981). Surgery for common epithelial tumours in the ovary. *Cancer*, **48**, 582.

WILS, J., BLIJHAM, G. & NAUS, A. (1986). Primary or delayed debulking surgery and chemotherapy consisting of *cis*-platin, doxorubicin, and cyclophosphamide in stage III–IV epithelial ovarian carcinoma. *J. Clin. Oncol.*, **4**, 1068.

ZIMMERMAN, P.V., BINT, M.H., HAWSON, G.A.T. & PARSONS, P.C. (1987). Ploidy as a prognostic determinant in surgically treated lung cancer. *Lancet*, **ii**, 530.